# Probiotic supplementation restores normal microbiota composition and function in antibiotic-treated and in caesarean-born infants

**DOI:** 10.1186/s40168-018-0567-4

**Published:** 2018-10-16

**Authors:** Katri Korpela, Anne Salonen, Outi Vepsäläinen, Marjo Suomalainen, Carolin Kolmeder, Markku Varjosalo, Sini Miettinen, Kaarina Kukkonen, Erkki Savilahti, Mikael Kuitunen, Willem M de Vos

**Affiliations:** 1 Immunobiology Research Programme, Department of Bacteriology and Immunology, University of Helsinki, Helsinki, Finland; 2 European Molecular Biology Laboratory, Heidelberg, Germany; 3 Department of Veterinary Biosciences, Faculty of Veterinary Medicine, University of Helsinki, Helsinki, Finland; 4 Department of Microbiome Science, Max Planck Institute for Developmental Biology, Tübingen, Germany; 5 Institute of Biotechnology, University of Helsinki, Helsinki, Finland; 6 Skin and Allergy Hospital, Department of Paediatrics, Helsinki University Hospital, Helsinki, Finland; 7 Children’s Hospital, University of Helsinki and Helsinki University Hospital, Helsinki, Finland; 8 Laboratory of Microbiology, Wageningen University, Wageningen, the Netherlands

**Keywords:** Early-life microbiota, Bifidobacteria, Lactobacilli, Metagenomics, Metaproteomics

## Abstract

**Background:**

Infants born by caesarean section or receiving antibiotics are at increased risk of developing metabolic, inflammatory and immunological diseases, potentially due to disruption of normal gut microbiota at a critical developmental time window. We investigated whether probiotic supplementation could ameliorate the effects of antibiotic use or caesarean birth on infant microbiota in a double blind, placebo-controlled randomized clinical trial. Mothers were given a multispecies probiotic, consisting of *Bifidobacterium breve* Bb99 (Bp99 2 × 10^8^ cfu) *Propionibacterium freundenreichii* subsp. *shermanii* JS (2 × 10^9^cfu), *Lactobacillus rhamnosus* Lc705 (5 × 10^9^ cfu) and *Lactobacillus rhamnosus* GG (5 × 10^9^ cfu) (*N* = 168 breastfed and 31 formula-fed), or placebo supplement (*N* = 201 breastfed and 22 formula-fed) during pregnancy, and the infants were given the same supplement. Faecal samples of the infants were collected at 3 months and analyzed using taxonomic, metagenomic and metaproteomic approaches.

**Results:**

The probiotic supplement had a strong overall impact on the microbiota composition, but the effect depended on the infant’s diet. Only breastfed infants showed the expected increase in bifidobacteria and reduction in *Proteobacteria* and *Clostridia*. In the placebo group, both birth mode and antibiotic use were significantly associated with altered microbiota composition and function, particularly reduced *Bifidobacterium* abundance. In the probiotic group, the effects of antibiotics and birth mode were either completely eliminated or reduced.

**Conclusions:**

The results indicate that it is possible to correct undesired changes in microbiota composition and function caused by antibiotic treatments or caesarean birth by supplementing infants with a probiotic mixture together with at least partial breastfeeding.

**Trial registration:**

clinicaltrials.gov NCT00298337. Registered March 2, 2006.

**Electronic supplementary material:**

The online version of this article (10.1186/s40168-018-0567-4) contains supplementary material, which is available to authorized users.

## Background

Massive microbial colonization of the human intestine begins at birth and is beginning to be understood as a finely tuned process [1, 2]. It is likely that infants are adapted to receiving specific microbial signals at critical time windows during their early development. The gut microbiota are emerging as a regulator of epigenetic programming [3] and critically influence the development of the immune system, with potentially irreversible effects on disease susceptibility [4, 5]. Furthermore, the gut microbiota have a central role in infant nutrition, affecting growth and energy metabolism [6]. Overall, gut microbiota development in early life likely contributes significantly to the long-term health of the host.

The natural bacterial colonization and development process is compromised when the infant is born by caesarean section [7–9], or given antibiotics [10, 11]. The early-life microbiota-disrupting factors have been found to be associated with later metabolic and immunological diseases, such as overweight [11–13], allergic disease [14, 15], type 1 diabetes [16] and inflammatory bowel disease [17, 18]. Mouse studies indicate that the early-life microbiota disruption plays a causal role in host phenotype development [19, 20]. Early exposure to antibiotics and birth by caesarean section are very common practices, affecting over 50% of infants in some populations [21, 22], making the issue of early-life microbiota disruption a significant public health concern.

While caesarean delivery and antibiotic treatment are medical necessities, it is becoming increasingly clear that preventing or reducing the disruptive effects on the microbiota is important for healthy infant development. However, thus far, there is very little evidence in support of specific treatment modalities. Lactobacilli and bifidobacteria hold great promise, as these bacteria are naturally an important component of the infant microbiota and have been found to reduce the risk of antibiotic-associated diarrhoea [1, 23]. A double-blinded placebo-controlled study in a cohort of over 1000 allergy-risk infants showed that the risk of allergic disease among caesarean-born infants could be reduced by pre- and postnatal supplementation of a multispecies probiotic, including *Bifidobacterium breve* Bb99 (Bp99 2 × 10^8^ cfu) *Propionibacterium freundenreichii* subsp. *shermanii* JS (2 × 10^9^ cfu), *Lactobacillus rhamnosus* Lc705 (5 × 10^9^ cfu) and *Lactobacillus rhamnosus* GG (5 × 10^9^ cfu) [24]. Here, we report the multiomics analysis of the intestinal microbiota in these infants and discover that the supplement effectively ameliorated most of the effects of caesarean birth and antibiotic use on infant microbiota.

## Results

### Supplement-induced microbiota-wide changes

**Fig. 1:**
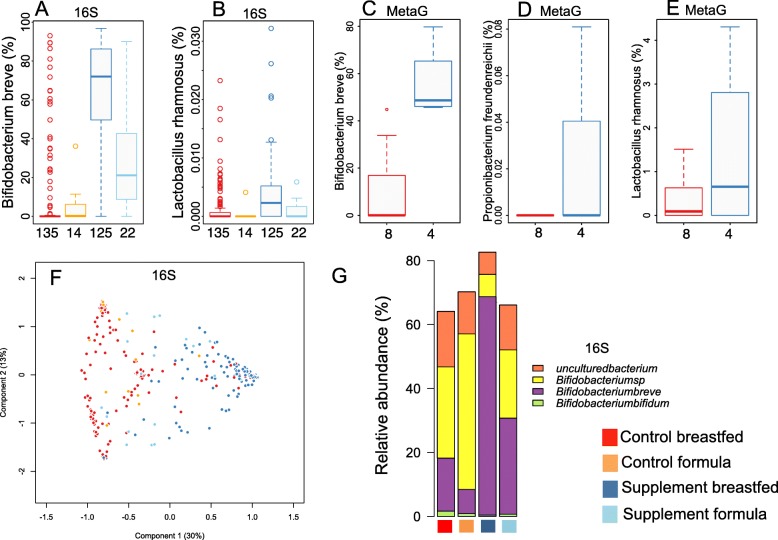
Effect of supplement treatment and feeding mode on the microbiota composition in the vaginally born, non-antibiotic-treated infants. **a**–**b** Relative abundance of the species in the probiotic mixture in 16S rRNA gene amplicon sequences derived from faecal samples. **c**–**e** Relative abundance of the probiotic species in whole metagenome sequences of breastfed infants. The number of infants per group is noted on the bottom of each panel (**a**–**e**). **f** Principal coordinates analysis (Bray-Curtis dissimilarities) on the species-level 16S rRNA gene data. **g** Composition of the *Bifidobacterium* population by treatment group and feeding type

We first investigated the effect of the intervention on the intestinal microbiota of vaginally born infants with no antibiotic treatments. The abundance of the gut-dwelling species present in the supplement, *Bifidobacterium breve* and *Lactobacillus rhamnosus*, were significantly increased in the supplemented group, up to over tenfold in the breast-fed but less in the formula-fed infants (Fig. 1a–b). The dominant species was *B. breve* in 84% of the breastfed infants and in 35% of the formula-fed infants in the supplemented group. In the control group, none of the formula-fed but 12% of the breastfed infants had *B. breve* as the dominant species, suggesting that *B. breve* is a natural colonizer of breastfed infants. To validate these findings with a PCR-independent approach, we also sequenced the whole metagenome of a subset of the breastfed infants’ faecal samples. This metagenomic analysis corroborated the phylogenetic approach, and the data captured also *Propionibacterium freundenreichii* present in the supplement (Fig. 1c–e), which was not identified in the 16S rRNA gene data.

The supplement affected the overall microbiota composition (Fig. 1f). Among the vaginally born infants with no antibiotic treatments, the main driver of inter-individual differences in the species-level intestinal microbiota composition was the treatment group, explaining 19% of the variation (*p* = 0.001 in permutational multivariate ANOVA, Fig. 1f). This result should be interpreted with caution due to the compositional nature of the data (with relative abundances summing up to 1). When inspecting the bifidobacterial community, we observed weaker responses to the supplement in the formula-fed compared to the breastfed infants (Fig. 1g).

**Fig. 2:**
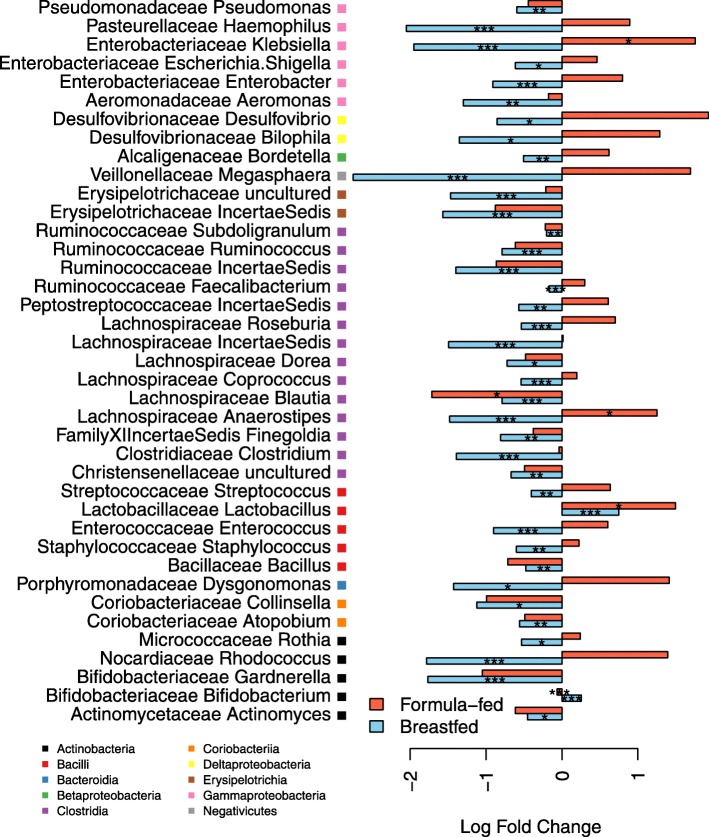
Effect of supplement treatment in the vaginally born, non-antibiotic-treated breastfed infants and formula-fed infants. The fold changes represent the difference in the relative abundance of the taxon between the supplement-treated group and the control group. The asterisks indicate the significance of the difference (based on GLM or GLS, see Additional file 1: Tables S1 and S2): *p < 0.05, **p < 0.01, ***p < 0.001

Further evidence of a mediating effect of feeding type came from analysis of specific bacterial taxa (Fig. 2): most of the supplement-induced changes observed in the breast-fed infants were not present in the formula-fed infants. In the breast-fed group, the abundance of lactobacilli was 100% (twofold) increased and that of bifidobacteria 29% increased in response to the supplement (*p* < 0.0001, generalized least squares model, GLS, Additional file 1: Table S1). Most other taxa were reduced in abundance (Fig. 2a, Additional file 1: Table S1): *Clostridia* by 66% (*p* < 0.0001, GLS) and *Gammaproteobacteria* by 58% (*p* < 0.0001, generalized linear model, GLM, with negative binomial distribution). In contrast, in the formula-fed infants, the total abundance of bifidobacteria was slightly but significantly decreased in the supplemented group (by 7%, *p* < 0.0001, GLM, Additional file 1: Table S2), despite the specific increase in *B. breve*. In addition, several *Firmicutes* and *Proteobacteria* taxa were increased in the formula-fed supplemented group compared to the formula-fed control group (Fig. 2): *Anaerostipe*s by fourfold (*p* = 0.05, GLM), *Veillonella* by sevenfold (*p* < 0.0001, GLM) and *Klebsiella* by sixfold (*p* = 0.05, GLM).

### Supplementation prevented caesarean birth-associated loss of bifidobacteria and normalized microbiota functions

**Fig. 3:**
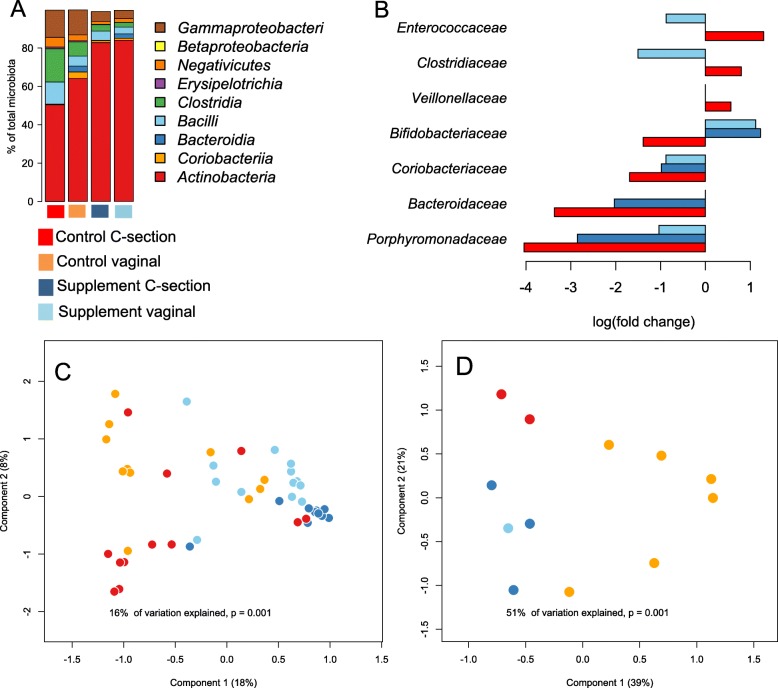
Effects of supplement and birth mode on the microbiota. Overall average composition of the microbiota at class level in the different groups, based on the 16S rRNA amplicon data (**a**). Significant family-level group differences compared to the vaginally born control group (**b**). Effect of supplement treatment and birth mode on the metaproteome (**c**) and metagenome (**d**) in principal coordinates analysis (Bray-Curtis dissimilarities)

After establishing a strong overall effect of the treatment, we tested if the supplement ameliorated some of the caesarean-induced changes in microbiota composition. In the control group, the microbiota of the caesarean-delivered infants was clearly different from the vaginally born infants (Fig. 3a), most notably due to a lower abundance of the most abundant genera, *Bifidobacterium* (75% decline, *p* = 0.01, GLS, Additional file 1: Table S3) and *Bacteroides* (96% decline, *p* < 0.0001, GLM). Overall, 6% (*p* = 0.001) of the inter-individual variation in microbiota composition was statistically attributable to birth mode in the control group according to permutational multivariate ANOVA. Remarkably, in the supplemented group, birth mode did not have a significant impact on microbiota composition (1% *p* = 0.08). The relative increases in *Enterococcaceae*, *Clostridiaceae* and *Veillonellaceae* that were observed in the section-born infants of the control group were not present in the supplemented group (Fig. 3b, Additional file 1: Table S3). Furthermore, the decline in *Bifidobacteriaceae* was prevented, and the declines in *Coriobacteriaceae*, *Porphyromonadaceae* and *Bacteroidaceae* were reduced in magnitude by the supplement (Fig. 3b, Additional file 1: Table S3).

The caesarean section-associated reduction of *Bifidobacterium* and *Bacteroides* spp., the two most important groups of bacteria in terms of carbohydrate degradation in infants, was reflected in the predicted carbohydrate degradation potential of the microbiota. Based on the taxonomic composition (abundance of taxa in the samples) and the CAZy database [25] (carbohydrate-active enzymes present in the taxa), the carbohydrate degradation potential in the samples was predicted. The predicted summarized total abundances of carbohydrate-active enzymes (CAZys) involved in the degradation of different types of carbohydrates, including human milk oligosaccharides, were significantly reduced among the caesarean-born infants (Additional file 2: Figure S1). The predicted abundances of these enzyme groups were increased in the supplemented infants, regardless of birth mode.

We conducted a metaproteome analysis in a subset of the cohort, including 11 vaginally born control infants, 12 caesarean-born control infants, 13 vaginally born supplemented infants and 12 caesarean-born supplemented infants. All were fully breastfed and had received no antibiotic treatments. The bacterial metaproteomes differed between birth modes in the control group, but were similar between birth modes in the supplemented group (Fig. 3c). Treatment with the supplement thus seemed to eliminate the effect of birth mode on the metaproteome-derived functions (Additional file 1: Table S4). This is illustrated by the finding that the supplemented groups (both caesarean-born and vaginal-delivered infants) compared to the vaginally born control group showed a high level of induction (up to 50-fold; Additional file 1: Table S4) of beta-galactosidase and beta-galactosyl *N*-acetyl hexosaminephosphorylase (LNBP), common bifidobacterial enzymes involved in the degradation of lactose and human milk oligosaccharides (HMOs), respectively [26]. In contrast, the bacteria in the caesarean-born infants expressed comparatively higher levels of aspartate aminotransferase and aspartate ammonia lyase, enzymes involved in potentially undesired protein degradation. The metaproteome data were also used to predict the taxonomic origin of the proteins, and the obtained results appeared similar to those from the 16S rRNA gene data (Additional file 2: Figure S2).

While the sample size in our metagenomic analysis was small, based on visual inspection of the PCoA plot, the genomic content of the microbiota appeared to cluster by birth mode and treatment: the metagenomes separated according to birth modes in the control group, but clustered together in the supplemented group (Fig. 3d). As the birth modes did not clearly differ in the supplemented group, we grouped together the faecal metagenomes of the supplemented infants for statistical analysis. Compared to the metagenomes in the vaginally born control group, a strong increase was observed for the lactose/galactose and rhamnose degradation genes (approximately 20-fold and 4-fold, respectively) while several amino acid and vitamin B (notably folic acid) synthesis pathways were significantly reduced (almost 30-fold) in the caesarean-born control group (Additional file 1: Table S5). These pathways were increased or unchanged in the supplemented group (Additional file 1: Table S5).

### Supplement prevented antibiotic-associated microbiota distortion

**Fig. 4:**
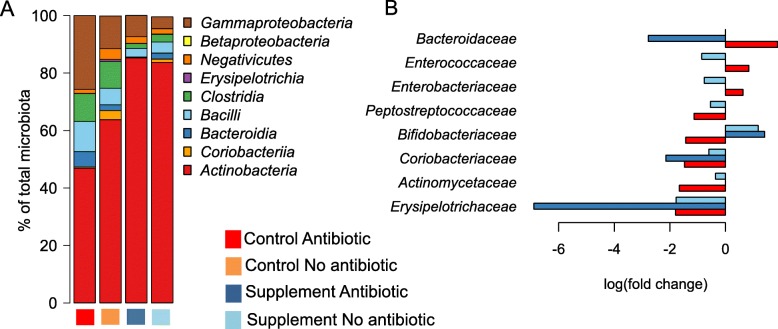
Effects of supplement treatment and antibiotic use on the microbiota composition in 16S rRNA amplicon data. Overall average composition of the microbiota at class level in the different groups (**a**). Significant family-level group differences compared to the non-antibiotic-treated control group (**b**)

Next, we assessed the effects of antibiotics on the microbiota and whether the supplement prevented these effects. In the control group, infants who had been treated with one or more antibiotic courses showed a clearly different microbiota composition compared to those that had received no antibiotics (Fig. 4a, Additional file 1: Table S6). In the control group, antibiotic use explained 4% (*p* = 0.001) of the microbiota composition, while in the treatment group antibiotic use did not have significant overall impact (< 1%, *p* = 0.56). In the control group, antibiotic use was associated with a decline in bifidobacteria by 17% (*p* = 0.015) and increases in *Enterococcus* and the Gram-negative classes *Gammaproteobacteria* and *Bacteroidia* by two-, two- and sixfold (*p* = 0.017, *p* = 0.04 and *p* < 0.0001), respectively. The supplement prevented or corrected the antibiotic-associated increases in *Bacteroidaceae*, *Enterococcaceae* and *Enterobacteriaceae* and the decline in *Bifidobacteriaceae* (Fig. 4b).

## Discussion

Using a combination of taxonomic, metagenomic and metaproteomic approaches, we showed that most of the antibiotic- and caesarean-associated changes in the faecal microbiota of infants could be corrected or reduced by a probiotic supplementation to mother and infant. The results indicate that breastfeeding together with the probiotic supplementation offer optimal results in terms of supporting the microbiota development in these infants. Both the mother and the infant received the same probiotic supplement. Based on the results, we cannot conclude what, if any, role the maternally ingested probiotic had on the infant microbiota.

Although the supplement contained oligosaccharides, the effect of the supplement on the microbiota composition was dependent on breastfeeding, suggesting that the amount of oligosaccharides in the supplement was insufficient to promote the probiotic strains. In fact, the relative abundance of bifidobacteria declined in the formula-fed supplemented group, indicating insufficient substrate availability to support the bifidobacterium community. Breast milk contains a diverse mixture of human milk oligosaccharides (HMOs), totaling approximately ca. 1 g/dl [27, 28], corresponding to a total intake of 5–13 g per day in an infant consuming between 5 and 13 dl of breast milk [29]. This is considerably more than the amount of galactooligosaccharides (0.8 g/day) in our study supplement. A dose-dependent bifidogenic effect of oligosaccharide supplementation ranging between 0.4–0.8 g/dl has previously been shown in formula-fed infants [30], and prebiotic supplementation at 0.8 g/dl has been shown to result in similar levels of bifidobacteria as full breastfeeding [31]. An amount of oligosaccharides close to those observed in breast milk are likely required to achieve the full bifidogenic effect. In addition to the amount of oligosaccharides, their composition may play a role. In this respect, HMOs offer a more diverse substrate mixture than galactooligosaccharides, and several strains of *B. breve* are able to utilize HMOs [32]. The results indicate that formula-fed infants may require additional supplementation of prebiotics that mimic the natural abundance of HMOs in breast milk to achieve the benefits of probiotics.

Remarkably, *L. rhamnosus* appeared to benefit from breastfeeding, even though it cannot utilize HMOs. The observed benefit of breastfeeding likely reflects a secondary effect of the increased abundance of bifidobacteria, rendering the conditions in the gut more favorable to *L. rhamnosus*. Bifidobacteria may have generated compounds from the HMOs that support growth of *L. rhamnosus*, such as fucose [33]. This suggests that probiotic products containing a synergistic mixture of bacteria may be more beneficial than single-strain products.

The most apparent effect of caesarean birth and antibiotic use was the relative reduction in bifidobacteria, which was corrected by the probiotic supplementation. As bifidobacteria are normally the most abundant bacterial group at this age, they contribute greatly to the overall microbial metabolic capacity. Thus, the supplement corrected or lessened most of the caesarean-associated changes in microbiota function. Caesarean-born infants in the control group had a microbiota with reduced capacity for carbohydrate degradation, importantly including the degradation of HMOs. Rather than degrading complex HMOs, the caesarean-associated microbiota seemed to focus on the utilization of lactose. The large amount of non-digestible oligosaccharides in breast milk promotes fermentation in the infant and hence the production of bacterial nutrients. Since the birth mode influences the composition of the gut microbes and their functional capacity, it is likely that the types and quantities of bacterial fermentation products vary between vaginally born and caesarean-born infants. Thus, the results indicate that caesarean birth may influence the composition of nutrients the infant receives from breast milk, possibly reducing the amount and altering the composition of SCFAs available to the host. Furthermore, several vitamin synthesis pathways were reduced in the section-born infants and corrected by the supplement. Folate in particular is important for healthy fetal and infant development [34] and is produced by certain strains of bifidobacteria, including *B. breve* strains [35]. Supplementation with folate-producing bifidobacterium strains and galactooligosaccharides, or with human milk, which increases the abundance of bifidobacteria, has been shown to increase serum folate levels in rats [36, 37]. Bifidobacteria may thus contribute a significant amount of folate available to a breastfed infant.

The low abundance of *Bacteroides*, which is ubiquitous in caesarean-born infants [7, 9, 38–43], was not fully corrected by the probiotic treatment. There is currently no known method of restoring the *Bacteroides* population in caesarean-born infants. Swabbing the infant with the mother’s vaginal fluids has been shown to fail at both *Bacteroides* and *Bifidobacterium* restoration [40], as these taxa do not normally occur in the vagina [44]. Furthermore, there are significant health risks involved in vaginal swabbing [45].

Despite the low abundance of *Bacteroides* spp. in the caesarean-born infants, the probiotic treatment was successful in reducing the incidence of allergic disease in this group [24], suggesting that the microbiota restoration achieved by the supplement was sufficient to induce a health benefit. This may be due to the normalization of the *Clostridium-Bifidobacterium* balance, as high abundance of *Clostridium* spp. and low abundance of bifidobacteria in infancy have been associated with increased risk of developing allergic disease [46, 47]. Furthermore, both *B. breve* Bb99 and *L. rhamnosus* GG are immunomodulatory bacteria, with anti-inflammatory effects [48, 49]. *L. rhamnosus* GG has been shown to elicit increased INF-γ production in infants with cow’s milk allergy [50] and to reduce atopic dermatitis symptoms in IgE-sensitized infants [51]. Both species have the capacity to bind to intestinal mucus [52]. Bifidobacteria isolated from healthy infant faeces, including *B. breve*, have been shown to have stronger mucus-binding capacity than bifidobacteria isolated from allergic infants, mostly *B. adolescentis* [52]. Adhesion to intestinal mucus makes direct interaction with host cells more likely and may be an important factor in early immune system education as well as providing colonization resistance and long-term mucosal colonization.

We have previously shown that *L. rhamnosus* GG supplementation for 7 months in preschool children prevented many penicillin/amoxicillin-associated changes in the microbiota, but failed to prevent the macrolide-associated loss of bifidobacteria [53]. Together with the present results, this indicates that optimal protection against antibiotic-associated microbiota disruption may be achieved by a mixture of *Bifidobacterium* and *Lactobacillus* strains. Indeed, studies in adults have shown that *Lactobacillus-Bifidobacterium* supplementation during and after *H. pylori* eradication therapy (amoxicillin-clarithromycin-lansopratzole) and during amoxicillin treatment helped to alleviate the antibiotic-associated microbiota disruption [54–56] and to reduce total gastrointestinal symptoms [57]. A prebiotic mixture containing 2.25 g fructo-oligosaccharides and inulin has been shown to increase the abundance of bifidobacteria and lactobacilli in amoxicillin-treated 1–2-year-old children [58].

The optimal duration of probiotic supplementation to prevent microbiota disruption has not been established. Since the microbiota imbalance in caesarean-born infants was restored by daily probiotic supplementation by the age of 3 months, it is possible that this is sufficient treatment duration. Previous studies on antibiotic-treated subjects have given daily probiotic/prebiotic supplements for 3 weeks during and after the antibiotic course with good results [54–58]. However, it may be beneficial to continuously supplement young infants with a probiotic mixture during the critical time of microbiota and immune system maturation.

## Conclusions

Early-life antibiotic treatments and caesarean birth influence a large fraction of the global population and are associated with global epidemic health problems, such as childhood overweight and immunological diseases. Therefore, treatments achieving even modest improvements at the level of individuals have the potential to induce great health benefits at the population level. Our results show that long-term daily *B. breve* and *L. rhamnosus* supplementation combined with breastfeeding is a safe and effective method to support the microbiota in caesarean-born and in antibiotic-treated infants. As these strains are already on the market, their use could be easily adopted into clinical practice.

## Methods

### Study design

We analyzed the gut microbiota composition using faecal samples and 16S rRNA gene amplicon sequencing in 428 infants at the age of 3 months comprising all high quality faecal samples of the cohort. A subset of the samples were additionally analyzed for whole metagenome and metaproteome composition. The infants were part of a probiotic trial (ClinicalTrials.gov Identifier: NCT00298337), the details of which have been published previously [24]. Pregnant mothers, whose infants had increased risk for allergy (at least one parent with a diagnosed allergic disease), were recruited at antenatal clinics and through advertisements in the Helsinki area in 1999–2000. A total of 1223 mothers were randomized in blocks of six at 35 weeks of gestation into a control group, receiving two daily capsules with microcrystalline cellulose, and a treatment group, receiving the same capsules once daily containing a mixture of cells (quantified as colony forming units, cfu) of *Bifidobacterium breve* Bb99 (Bp99 2 × 10^8^ cfu), *Propionibacterium freundenreichii* subsp. *shermanii* JS (2 × 10^9^cfu), *Lactobacillus rhamnosus* Lc705 (5 × 10^9^ cfu) and *Lactobacillus rhamnosus* GG (5 × 10^9^ cfu) until birth. For the first 6 months following birth, the infants received the same capsules that were opened and mixed into sugar syrup, which in the treatment group was further supplemented with 0.8 g galactooligosaccharides (GOS). Adverse effects were not encountered in either group. Randomization was done by a statistician, and allocation was concealed from study participants, care givers, all study doctors and nurses. Blinding was kept until after the 5-year evaluation had been done. Faecal samples were collected from the infants at 3 months of age and stored at − 40 °C. For the present study, faecal samples were available from 428 infants (Table 1). The parents provided information on birth mode, breastfeeding duration, use of formula feeding and antibiotic use via questionnaires.

**Table 1:** Characteristics of the cohort, excluding the six infants with insufficient sequencing reads (< 100 reads)

	Control breastfed	Control formula-fed	Supplement breastfed	Supplement formula-fed	Total *N*
Total *N*	201	22	168	31	422
Caesarean	39 (19%)	5 (23%)	28 (16%)	7 (23%)	79 (19%)
Antibiotics	27 (13%)	3 (14%)	15 (9%)	2 (7%)	47 (11%)

### Sample processing

DNA was extracted from the faecal samples using a repeated bead-beating protocol [59], and the bacterial composition was analyzed by Illumina MiSeq sequencing the hypervariable V3-V4 region of the 16S rRNA gene. The library preparation was performed essentially according to the protocol by Illumina (https://support.illumina.com/content/dam/illumina-support/documents/documentation/chemistry_documentation/16s/16s-metagenomic-library-prep-guide-15044223-b.pdf) except that the 16S rRNA gene amplification and barcoding were performed in a single reaction. The PCR reaction comprised of 1 ng/μl template, 1X Phusion® Master Mix (ThermoFisher, catalog number: F-531 L), 0.25 μM V3-V4 locus-specific primers and 0.375 μM TruSeq dual-index primers. The PCR was run under the following settings: 98 °C for 30 s, 27 cycles of 98 °C for 10 s, 62 °C for 30 s, 72 °C for 15 s and finally 10 min at 72 °C, where after the samples were stored at 4 °C. The PCR clean-up was performed with AMPure XP beads (Beckman Coulter, Copenhagen, Denmark), and confirmation of the correct amplicon size (ca. ~ 640 base pairs) was performed on a Bioanalyzer DNA 1000 chip (Agilent Technology, Santa Clara, CA, USA). The randomly pooled libraries were sequenced with an Illumina MiSeq or HiSeq 2500 in Rapid Run mode.

We further analyzed the metagenomes of a small subset of infants, including six vaginally born control infants, two control infants born by C-section, one vaginally born and three C-section-born infants in the treatment group. All were fully breastfed and had no antibiotic treatments. For metagenomic analysis, the selected DNA preps were purified with the DNA Clean & Concentrator TM-5 Kit (Zymo Research, Ontario, Canada) and eluted in low-TE buffer after which the libraries were prepared using Nextera DNA kit and sequenced in HiSeq Rapid SE200–run using 50% of the flow cell.

Selected faecal samples (*n* = 48) were subject to metaproteome analysis by processing an aliquot (125 mg) of the freshly thawed faecal samples and extracting the proteins by bead beating as described previously [60]. Protein analysis and the subsequent peptide identification of bacterial proteins were performed as described previously [61]. Proteins were subject to denaturing SDS-polyacrylamide gel electrophoresis to remove impurities, and proteins with an expected subunit size of 5–500 kD were recovered from the gel, alkylated and trypsin digested as described previously. Finally, the protein digests were analyzed by LC-MS/MS on a nanoflow HPLC system (Easy-nLCII, Thermo Fisher Scientific) coupled to a LTQ Orbitrap Elite mass spectrometer (Thermo Fisher Scientific, Bremen, Germany) equipped with a nano-electrospray ionization source. This and the subsequent peptide identification of bacterial were performed essentially as described previously [61].

### Statistical analysis

The 16S rRNA amplicon sequencing reads were analyzed using the R package mare [62]. The median number of reads obtained per sample was 46,934 and varied from 105 to 151,840. Samples with < 100 reads (*N* = 6) were excluded from the analysis, as they seemed to have insufficient coverage based on the estimated richness. Although paired-end sequencing was conducted, we only used the forward reads truncated to 150 bases, as we have observed using artificial communities of known composition that longer reads provide unreliable results [62]. Potential sequencing errors were removed by discarding unique reads, which occurred < 100 times in the total dataset. Taxonomic annotation was performed using USEARCH [63] by mapping the reads to the SILVA 16S rRNA reference database version 115 [64], restricted to gut-associated taxa. The metagenomic sequences were quality filtered using USEARCH and then analyzed using HUMAnN2 and Metaphlan2 [65].

The statistical analysis was conducted in R with the package mare [62], with tools from packages vegan [66], MASS [67] and nlme [68]. Based on the taxonomic data, we predicted the carbohydrate metabolism capacity of the microbiota using the CAZy database [25]. Associations between the overall microbiota composition and background variables were assessed using principal coordinates analysis and multivariate permutational analysis of variance. For assessing the effect of birth mode, the infants were divided into four groups based on treatment and birth mode, using the vaginally born control group as the reference group, and the models were adjusted for antibiotic use and feeding type. When assessing the effect of antibiotic use, the infants were divided into four groups based on treatment and antibiotic use, using the non-antibiotic-treated control group as the reference group, and the models were adjusted for birth mode and feeding type. The effect of the treatment, antibiotic exposure, caesarean birth and feeding type (full or partial breastfeeding or exclusive formula feeding) on the abundance of the bacterial taxa were analyzed using negative binomial models, with the number of reads per sample as the offset. If the fitted model failed to fulfill model assumptions (primarily heteroscedasticity of the residuals), the observed problems were corrected using generalized least squares models. For this reason, the statistical test is not the same for all taxa, as the distribution of the data varies between taxa. Only genera observed in > 10% of the samples were analyzed individually.

We included a set of negative control samples, consisting of only PCR reagents, and sequenced these together with the real samples. Overall, the number of reads for the negative controls was very small (median 260 reads) compared to the real samples (median 46,674 reads). This indicates that contaminants likely contributed only a few hundred reads per sample, which would not greatly influence the overall observed composition. The most abundant taxa in the negative controls were *Pseudomonas* and *Rhodococcus*, which formed a very small abundance in the real samples, representing respectively 0.2% and 1.2% of total reads on average. In addition, we have previously published data on positive control samples (mock communities), validating the sequencing and bioinformatics methods [69].

## Additional files

Additional file 1: Model results. (XLSX 153 kb)

Additional file 2: **Figure S1.** Significant differences between groups defined by birth mode and supplement treatment on the predicted carbohydrate-active enzyme abundance. Vaginally born control group is the reference group to which other groups (red = section-born control, dark blue = section-born supplemented, light blue = vaginally born supplemented) are compared. Non-significant (*p* > 0.05) differences are set to 0. **Figure S2.** Comparison of the observed differences in relative abundance of bacterial genera in the control-caesarean, probiotic-caesarean and probiotic-vaginal groups to the reference group, control-vaginal. The bars (solid = 16S rRNA data; dashed = metaproteome data) indicate the magnitude of the difference (log fold change) and the asterisks indicate the level of significance: ***p < 0.001; **p < 0.01; *p < 0.05. (DOCX 2428 kb))

## References

[1] Milani C, Duranti S, Bottacini F, Turroni F, Mahony J, et al. The first microbial colonizers of the human gut: composition, activities, and health implications of the infant gut microbiota. Microbiol Mol Biol Rev. 2017;81(4). 10.1128/MMBR.00036-17. 10.1128/MMBR.00036-17 PMC5706746 29118049

[2] Korpela K, Costea P, Coelho LP, Kandels-Lewis S, Willemsen G, Boomsma DI (2018). Selective maternal seeding and environment shape the human gut microbiome. Genome Res.

[3] Krautkramer KA, Kreznar JH, Romano KA, Vivas EL, Barret-Wilt GA, Rabaglia ME (2016). Diet-microbiota interactions mediate global epigenetic programming in multiple host tissues. Mol Cell.

[4] Maynard CL, Elson CO, Hatton RD, Weaver CT (2012). Reciprocal interactions of the intestinal microbiota and immune system. Nature.

[5] Gensollen T, Iyer SS, Kasper DL, Blumberg RS (2016). How colonization by microbiota in early life shapes the immune system. Science.

[6] Blanton LV, Charbonneau MR, Salih T, Barratt MJ, Venkatesh S, Ilkaveya O (2016). Gut bacteria that prevent growth impairments transmitted by microbiota from malnourished children. Science.

[7] Biasucci G, Rubini M, Riboni S, Morelli L, Bessi E, Retetangos C (2010). Mode of delivery affects the bacterial community in the newborn gut. Early Hum Dev.

[8] Dominguez-Bello MG, Costello EK, Contreras M, Magris M, Hidalgo G, Fierer N (2010). Delivery mode shapes the acquisition and structure of the initial microbiota across multiple body habitats in newborns. Proc Natl Acad Sci.

[9] Bäckhed F, Roswall J, Peng Y, Feng Q, Jia H, Kovatcheva-Datchary P (2015). Dynamics and stabilization of the human gut microbiome during the first year of life. Cell Host Microbe.

[10] Persaud R, Azad B, Konya T, Guttman D, Chari R, Sears M (2014). Impact of perinatal antibiotic exposure on the infant gut microbiota at one year of age. Allergy Asthma Clin Immunol.

[11] Korpela K, Salonen A, Virta LJ, Kekkonen RA, de Vos WM (2016). Association of early-life antibiotic use and protective effects of breastfeeding: role of the intestinal microbiota. JAMA Pediatr.

[12] Mueller NT, Whyatt R, Hoepner L, Oberfield S, Dominguez-Bello MG, Widen EM (2015). Prenatal exposure to antibiotics, cesarean section and risk of childhood obesity. Int J Obes.

[13] Saari A, Virta LJ, Sankilampi U, Dunkel L, Saxen H (2015). Antibiotic exposure in infancy and risk of being overweight in the first 24 months of life. Pediatrics.

[14] Bager P, Wohlfahrt J, Westergaard T (2008). Caesarean delivery and risk of atopy and allergic disesase: meta-analyses. Clin Exp Allergy.

[15] van Nimwegen FA, Penders J, Stobberingh EE, Postma DS, Koppelman GH, Kerkhof M (2011). Mode and place of delivery, gastrointestinal microbiota, and their influence on asthma and atopy. J Allergy Clin Immunol.

[16] Cardwell C, Stene L, Joner G, Cinek O, Scensson J, Goldacre MJ (2008). Caesarean section is associated with an increased risk of childhood-onset type 1 diabetes mellitus: a meta-analysis of observational studies. Diabetologia.

[17] Hviid A, Svanstrom H, Frisch M (2011). Antibiotic use and inflammatory bowel diseases in childhood. Gut.

[18] Virta L, Auvinen A, Helenius H, Huovinen P, Kolho K (2012). Association of repeated exposure to antibiotics with the development of pediatric Crohn’s disease-a nationwide, register-based Finnish case-control study. Am J Epidemiol.

[19] Russell SL, Gold MJ, Hartmann M, Willing BP, Thorson L, Wlodarska M (2012). Early life antibiotic-driven changes in microbiota enhance susceptibility to allergic asthma. EMBO Rep.

[20] Cox LM, Yamanishi S, Sohn J, Alekseyenko AV, Leung JM, Cho I (2014). Altering the intestinal microbiota during a critical developmental window has lasting metabolic consequences. Cell.

[21] Bergus GR, Levy BT, Levy SM, Slager SL, Kiritsy MC (1996). Antibiotic use during the first 200 days of life. Arch. Fam. Med.

[22] Betrán AP, Ye J, Moller AB, Zhang J, Gülmezoglu AM, Torloni MR (2016). The increasing trend in caesarean section rates: global, regional and national estimates: 1990-2014. PLoS One.

[23] Corrêa NB, Péret Filho LA, Penna FJ, Lima FMS, Nicoli JR (2005). A randomized formula controlled trial of Bifidobacterium lactis and Streptococcus thermophilus for prevention of antibiotic-associated diarrhea in infants. J Clin Gastroenterol.

[24] Kuitunen M, Kukkonen K, Juntunen-Backman K, Korpela R, Poussa T, Tuure T (2009). Probiotics prevent IgE-associated allergy until age 5 years in cesarean-delivered children but not in the total cohort. J Allergy Clin Immunol.

[25] Lombard V, Golaconda Ramulu H, Drula E, Coutinho PM, Henrissat B (2013). The carbohydrate-active enzymes database (CAZy) in 2013. Nucleic Acids Res.

[26] James K, Motherway MO, Bottacini F, Van Sinderen D (2016). Bifidobacterium breve UCC2003 metabolises the human milk oligosaccharides lacto-N-tetraose and lacto-N-neo-tetraose through overlapping, yet distinct pathways. Sci Rep.

[27] Coppa GV, Gabrielli O, Pierani P, Catassi C, Carlucci A, Giorgi PL (1993). Changes in carbohydrate composition in human milk over 4 months of lactation. Pediatrics.

[28] Thurl S, Müller-Werner B, Sawatzki G (1996). Quantification of individual oligosaccharide compounds from human milk using high-pH anion-exchange chromatography. Anal Biochem.

[29] Kent JC, Mitoulas LR, Cregan MD, Ramsay DT, Doherty DA, Hartmann PE (2006). Volume and frequency of breastfeedings and fat content of breast milk throughout the day. Pediatrics.

[30] Moro G, Minoli I, Mosca M, Fanaro S, Jelinek J, Stahl B (2002). Dosage-related bifidogenic effects of galacto-and fructooligosaccharides in formula-fed term infants. J Pediatr Gastroenterol Nutr.

[31] Rinne M, Gueimonde M, Kalliomäki M, Hoppu U, Salminen SJ, Isolauri E (2005). Similar bifidogenic effects of prebiotic-supplemented partially hydrolyzed infant formula and breastfeeding on infant gut microbiota. FEMS Immunol Med Microbiol.

[32] Ruiz-Moyano S, Totten S, Garrido D, Smilowitz J, German J, Lebrilla C (2013). Variation in consumption of human milk oligosaccharides by infant gut-associated strains of Bifidobacterium breve. Appl. Environ. Microbiol.

[33] Douillard FP, Ribbera A, Kant R, Pietilä T, Järvinen H, Messing M (2013). Comparative genomic and functional analysis of 100 Lactobacillus rhamnosus strains and their comparison with strain GG. PLoS Genet.

[34] Molloy AM, Kirke PN, Brody LC, Scott JM, Mills JL (2008). Effects of folate and vitamin B12 deficiencies during pregnancy on fetal, infant, and child development. Food Nutr Bull.

[35] Deguchi Y, Morishita T, Mutai M (1985). Comparative studies on synthesis of water-soluble vitamins among human species of bifidobacteria. Agric Biol Chem.

[36] Krause LJ, Forsberg CW, Connor DL (1996). Feeding human milk to rats increases Bifidobacterium in the cecum and colon which correlates with enhanced folate status. J Nutr.

[37] Pompei A, Cordisco L, Amaretti A, Zanoni S, Raimondi S, Matteuzzi D (2007). Administration of folate-producing bifidobacteria enhances folate status in Wistar rats. J Nutr.

[38] Penders J, Thijs C, Vink C, Stelma FF, Snijders B, Kummeling I (2006). Factors influencing the composition of the intestinal microbiota in early infancy. Pediatrics.

[39] Penders J, Gerhold K, Stobberingh EE, Thijs C, Zimmermann K, Lau S (2013). Establishment of the intestinal microbiota and its role for atopic dermatitis in early childhood. J Allergy Clin Immunol.

[40] Dominguez-Bello MG, Jesus-laboy K, Shen N, Cox L, Amir A, Gonzalez A (2016). Partial restoration of the microbiota of cesarean-born infants via vaginal microbial transfer. Nat. Med.

[41] Madan JC, Hoen AG, Lundgren SN, Farzan SF, Cottingham KL, Morrison HG (2016). Association of cesarean delivery and formula supplementation with the intestinal microbiome of 6-week-old infants. JAMA pediatrics.

[42] Chu DM, Ma J, Prince AL, Antony KM, Seferovic MD, Aagaard KM (2017). Maturation of the infant microbiome community structure and function across multiple body sites and in relation to mode of delivery. Nat Med.

[43] Sordillo JE, Zhou Y, McGeachie MJ, Ziniti J, Lange N, Laranjo N (2017). Factors influencing the infant gut microbiome at age 3-6 months: findings from the ethnically diverse vitamin D antenatal asthma reduction trial (VDAART). J Allergy Clin Immunol.

[44] Ravel J, Gajer P, Abdo Z, Schneider GM, Koenig SS, McCulle SL (2011). Vaginal microbiome of reproductive-age women. Proc Natl Acad Sci U S A.

[45] Cunnington AJ, Sim K, Deierl A, Kroll J, Brannigan E, Darby J (2016). “Vaginal seeding” of infants born by caesarean section. BMJ.

[46] Björkstén B, Sepp E, Julge K, Voor T, Mikelsaar M (2001). Allergy development and the intestinal microflora during the first year of life. J Allergy Clin Immunol.

[47] Kalliomäki M, Kirjavainen P, Eerola E, Kero P, Salminen S, Isolauri E (2001). Distinct patterns of neonatal gut microflora in infants in whom atopy was and was not developing. J Allergy Clin Immunol.

[48] Li N, Russell WM, Douglas-esobar M, Hauser N, Lopez M, Neu J (2009). Live and heat-killed Lactobacillus rhamnosus GG: effects on proinflammatory and anti-inflammatory cytokines/chemokines in gastrostomy-fed infant rats. Pediatr Res.

[49] Jeon SG, Kayama H, Ueda Y, Takahashi T, Asahara T, Tsuji H (2012). Probiotic Bifidobacterium breve induces IL-10-producing Tr1 cells in the colon. PLoS Pathog.

[50] Pohjavuori E, Viljanen M, Korpela R, Kuitunen M, Tiittanen M, Vaarala O (2004). Lactobacillus GG effect in increasing IFN-γ production in infants with cow’s milk allergy. J Allergy Clin Immunol.

[51] Viljanen M, Savilahti E, Haahtela T, Juntunen-Backman K, Korpela R, Poussa T (2005). Probiotics in the treatment of atopic eczema/dermatitis syndrome in infants: a double-blind placebo-controlled trial. Allergy.

[52] He F, Ouwehand A, Isolauri E, Hashimoto H, Benno Y, Salminen S (2001). Comparison of mucosal adhesion and species identification of bifidobacteria isolated from healthy and allergic infants. FEMS Immunol Med Microbiol.

[53] Korpela K, Salonen A, Virta L, Kumpu M, Kekkonen R, de Vos W (2016). *Lactobacillus* rhamnosus GG intake modifies preschool Children’s intestinal microbiota, alleviates penicillin-associated changes, and reduces antibiotic use. PLoS One.

[54] Plummer SF, Garaiova I, Sarvotham T, Cottrall SL, Le Scouiller, Weaver MA (2005). Effects of probiotics on the composition of the intestinal microbiota following antibiotic therapy. Int J Antimicrob Agents.

[55] Myllyluoma E, Ahlroos T, Veijola L, Rautelin H, Tynkkynen S, Korpela R (2007). Effects of anti-helicobacter pylori treatment and probiotic supplementation on intestinal microbiota. Int J Antimicrob Agents.

[56] Engelbrektson A, Korzenik JR, Pittler A, Sanders ME, Klaenhammer TR, Leyer G (2009). Probiotics to minimize the disruption of faecal microbiota in healthy subjects undergoing antibiotic therapy. J Med Microbiol.

[57] Myllyluoma E, Veijola L, Ahlroos T, Tynkkynen S, Kankuri E, Vapaatalo H (2005). Probiotic supplementation improves tolerance to helicobacter pylori eradication therapy–a placebo-controlled, double-blind randomized pilot study. Aliment. Pharmacol. Ther.

[58] Brunser O, Gotteland M, Cruchet S, Figueroa G, Garrido D, Steenhout P (2006). Effect of a milk formula with prebiotics on the intestinal microbiota of infants after an antibiotic treatment. Pediatr Res.

[59] Salonen A, Nikkilä J, Jalanka-Tuovinen J, Immonen O, Rajilić-Stojanović M, Kekkonen RA (2010). Comparative analysis of fecal DNA extraction methods with phylogenetic microarray: effective recovery of bacterial and archaeal DNA using mechanical cell lysis. J Microbiol Methods.

[60] Kolmeder CA, de Been M, Nikkilä J, Ritamo I, Mättö J, Valmu L (2010). Comparative metaproteomics and diversity analysis of human intestinal microbiota testifies for its temporal stability and expression of core functions. PLoS One.

[61] Kolmeder CA, Salojärvi J, Ritari J, de Been M, Raes J, Falony G (2016). Faecal metaproteomic analysis reveals a personalized and stable functional microbiome and limited effects of a probiotic intervention in adults. PLoS One.

[62] Korpela, K. mare: Microbiota Analysis in R Easily. R package version 1.0. 2016.

[63] Edgar RC (2010). Search and clustering orders of magnitude faster than BLAST. Bioinformatics.

[64] Quast C, Pruesse E, Yilmaz P, Gerken J, Schweer T, Yarza P (2012). The SILVA ribosomal RNA gene database project: improved data processing and web-based tools. Nucleic Acids Res.

[65] Truong DT, Franzosa EA, Tickle TL, Scholz M, Weingart G, Pasolli E (2015). MetaPhlAn2 for enhanced metagenomic taxonomic profiling. Nat Methods.

[66] Oksanen, J. *et al*. vegan: Community Ecology Package. R package version 2.0-6. 2013.

[67] Venables W, Ripley B (2002). Modern Applied Statistics with S.

[68] Pinheiro, J., Bates, D., DebRoy, S., Sarkar, D. & the R Development Core Team. nlme: Linear and Nonlinear Mixed Effects Models. R package version 3.1-108. 2013.

[69] Korpela K, Blakstad EW, Moltu S, Strommen K, Nakstad B, Ronnestad (2018). Intestinal microbiota development and gestational age in preterm neonates. Sci Rep.

