# Supplementary material for: Probiotic supplementation restores normal microbiota composition and function in antibiotic-treated and in caesarean-born infants
Source: Microbiome. 2018 Oct 16;6:182. doi: 10.1186/s40168-018-0567-4 (PMC6192119; doi:10.1186/s40168-018-0567-4)
Supplement: Supplementary file 2 — Figure S1. Significant differences between groups defined by birth mode and supplement treatment on the predicted carbohydrate-active enzyme abundance. Vaginally born control group is the reference group to which other groups (red = section-born control, dark blue = section-born supplemented, light blue = vaginally born supplemented) are compared. Non-significant (p > 0.05) differences are set to 0. Figure S2. Comparison of the observed differences in relative abundance of bacterial genera in the control-caesarean, probiotic-caesarean and probiotic-vaginal groups to the reference group, control-vaginal. The bars (solid = 16S rRNA data; dashed = metaproteome data) indicate the magnitude of the difference (log fold change) and the asterisks indicate the level of significance: ***p < 0.001; **p < 0.01; *p < 0.05. (DOCX 2428 kb)) [file 40168_2018_567_MOESM2_ESM.docx]

Additional file 1

**Probiotic supplementation restores normal microbiota composition and function in antibiotic-treated and in caesarean-born infants**

**Figure S1**. Significant differences between groups defined by birth mode and supplement treatment on the predicted carbohydrate-active enzyme abundance. Vaginally born control group is the reference group to which other groups (red = section-born control, dark blue = section-born supplemented, light blue = vaginally born supplemented) are compared. Non-significant (p>0.05) differences are set to 0.

**Figure S2**. Comparison of the observed differences in relative abundance of bacterial genera in the control-caesarean, probiotic-caesarean, and probiotic-vaginal groups to the reference group, control-vaginal. The bars (solid = 16S rRNA data; dashed = metaproteome data) indicate the magnitude of the difference (log fold change) and the asterisks indicate the level of significance: *** p<0.001; ** p<0.01; * p<0.05.
